# Preparedness and response activities of the US Department of Veterans Affairs (VA) home-based primary care program around the fall 2017 hurricane season

**DOI:** 10.1186/s12889-020-09888-8

**Published:** 2020-11-26

**Authors:** Tamar Wyte-Lake, Claudia Der-Martirosian, Karen Chu, Rachel Johnson-Koenke, Aram Dobalian

**Affiliations:** 1 Veterans Emergency Management Evaluation Center (VEMEC), U.S. Department of Veterans Affairs, 16111 Plummer St. MS-152, North Hills, CA 91343 USA; 2 Department of Family Medicine, Oregon Health & Science University, 3181 SW Sam Jackson Park Rd, Portland, OR 97239 USA; 3 Denver-Seattle Center of Innovation, Rocky Mountain Regional VA Medical Center, U.S. Department of Veterans Affairs, 1700 North Wheeling Street, Aurora, CO 80045-7211 USA; 4 Division of Health Systems Management and Policy, University of Memphis School of Public Health, 3720 Alumni Ave, Memphis, TN 38152 USA

**Keywords:** Emergency preparedness, Home health agencies, Long-term care, Aging in place

## Abstract

**Background:**

Large-scale natural disasters disproportionally affect both the medically complex and the older old, groups that are responsible for most medical surge after a disaster. To understand how to ameliorate this surge, we examined the activities of the nine US Department of Veterans Affairs (VA) Home Based Primary Care (HBPC) programs impacted during the 2017 Fall Hurricane Season.

**Methods:**

Convergent mixed methods design, incorporating independently conducted qualitative and quantitative analyses. Phase One: 34 clinical staff were interviewed from the nine VA HBPC programs impacted by Hurricanes Harvey, Irma, and Maria to examine the experiences of their HBPC programs in response to the Hurricanes. Phase Two: Secondary quantitative data analysis used the VA’s Corporate Data Warehouse (CDW) to examine the electronic health records of patients for these same nine sites.

**Results:**

The emergency management activities of the HBPC programs emerged as two distinct phases: preparedness, and response and recovery. The early implementation of preparedness procedures, and coordinated post-Hurricane patient tracking, limited disruption in care and prevented significant hospitalizations among this population.

**Conclusions:**

Individuals aged 75 or older, who often present with multiple comorbidities and decreased functional status, typically prefer to age in their homes. Additionally, as in-home medical equipment evolves, more medically vulnerable individuals are able to receive care at home. HBPC programs, and similar programs under Medicare, connect the homebound, medically complex, older old to the greater healthcare community. Engaging with these programs both pre- and post-disasters is central to bolstering community resilience for these at-risk populations.

**Supplementary Information:**

The online version contains supplementary material available at 10.1186/s12889-020-09888-8.

## Background

Within the next two decades, the number of households led by someone aged seventy-five years of age or older will double to 28.2 million [1]. Notwithstanding the fact that the majority of these individuals present with multiple co-morbidities and decreased activities of daily living [2], these individuals want to age in their homes and communities [3, 4]. Further, as in-home medical equipment evolves, there is a growing percentage of the medically vulnerable population who can receive care at home rather than in a healthcare facility. For example, more than 2.5 million Medicare beneficiaries rely on electricity-dependent medical equipment, such as ventilators, to live independently in their homes [5]. Concomitantly, there is an increasing frequency of large-scale natural disasters [6]. These disasters disproportionally affect both the medically complex [7] and the older old [8, 9]. Consequently, it is incumbent on communities to mitigate the impact of disasters on community-dwelling, medically vulnerable older adults.

Home health agencies (HHAs) provide supportive healthcare services to community-dwelling, medically vulnerable older adults. Medicare certified HHAs provide skilled, multi-disciplinary healthcare in the home, and the number of HHAs across the United States is growing [10]. Accordingly, the new Centers for Medicare and Medicaid Services (CMS) Emergency Preparedness Rule included HHAs among the 17 provider and supplier types that are required to meet national emergency preparedness standards for both natural and manmade disasters [11]. Yet, there is limited understanding of the actual efforts required for these programs to implement a preparedness, response, and recovery program in an actual disaster event.

Home Based Primary Care is a subset of home health care. The CMS Independence at Home (IAH) demonstration has supported a growing interest in understanding the benefits of expanding this model of care [12–14]. The Veterans Health Administration (VA) Home Based Primary Care (HBPC) Program, which has a similar model to the IAH demonstration [14, 15], and has been in practice for more than 40 years, serves a population with a mean age of 76.5 years. Like a traditional HHA, VA HBPC provides interdisciplinary care in the home to select veterans who present with complex chronic disease [16]. To understand the degree of support required for home-based care programs to support their staff and patients through a natural disaster, we conducted a mixed methods study of the VA’s HBPC programs impacted by the Fall 2017 Hurricanes (Hurricane Harvey (August 2017), Hurricanes Irma and Maria (September 2017).

## Methods

This study was part of a larger study that aimed to understand the role of VA HBPC during the Fall 2017 Hurricane season [17]. To obtain a multifaceted understanding of support provided by HBPC programs to support staff and their patients through a natural disaster, we applied a convergent mixed methods design, whereby we incorporated independently conducted qualitative and quantitative analyses. Using a mixed-methods design allowed the exploration of both qualitative insights of program staff directly involved in the hurricane preparedness and response, as well as a quantitative illustration of the timeline of activities around the hurricanes at the same sites. Phase One of this study used qualitative interviews to examine the experiences of nine VA HBPC programs in their responses to Hurricanes Harvey, Irma, and Maria (Fall 2017 Hurricanes). Phase Two of this study used the VA’s Corporate Data Warehouse (CDW) to quantitatively examine HBPC data for these same nine sites.

### Qualitative data

#### Study design

The first part of the study used qualitative interviews with key stakeholders from HBPC programs located in regions impacted by Hurricanes Harvey, Irma, and Maria (Fall 2017 Hurricanes) in order to explore their experiences as part of their program’s Hurricane response.

#### Setting

We studied VHA HBPC programs impacted by the Fall 2017 Hurricanes. The HBPC program is composed of an interdisciplinary team. The clinical staff are generally composed of nurse practitioners, registered nurses, social workers, occupational therapists, and dieticians, as well as physicians, physical therapists, and psychologists. Each VA Medical Center (VAMC) has an HBPC program. The HBPC programs can also be located in an affiliated Community Based Outpatient Clinic (CBOC), where the HBPC clinical staff are based at the CBOC, but they share a Program Director with the main VAMC. At eight of the nine sites the main VA Medical Center (VAMC) was impacted by a hurricane. At three sites, an affiliated CBOC was more seriously impacted than the primary facility, while at the other six sites, both the main VAMC and multiple affiliated CBOCs were impacted.

#### Sample

Nine VA HBPC programs were included from Texas, Florida and Puerto Rico. We used a purposive sampling approach, with the HBPC Program Director as the first point of contact at each site. These individuals were then asked to identify additional HBPC team members who were on staff at the time of the Hurricanes. Study staff then contacted these additional team members by email and phone. All respondents were reminded that study participation was voluntary. We conducted 34 interviews. Due to minimal impact by a hurricane, it was determined that saturation was reached at one site after speaking to a single respondent. All other sites had three to five respondents.

#### Design and data collection methods

We developed semi-structured interview guides (see Supplementary file 1) that queried respondents on three key themes: hurricane preparedness protocols and activities, hurricane response activities, with a focus on continuity of care, and facilitators and barriers to disaster response. Respondents were asked to describe their role in the HBPC program and any past experiences with disasters. Participants were interviewed separately by phone for 30 to 60 min, and interviews were audiotaped and transcribed. At one site, a focus group with clinical staff was conducted due to scheduling conflicts. In this case, all participants agreed to the joint sharing of experiences.

#### Analysis plan

All interview data were uploaded into Atlas.ti (v.7) for analysis. The initial code list was composed of an a priori code list, which was then revised an expanded, based on in-vivo coding methods [18]. All interviews were coded independently by authors XX and XX., All disagreements were resolved by consensus.

### Quantitative data

Using data from the VA CDW, a national repository of clinical and administrative data from VA medical facilities, information about each clinical visit was extracted for the three most impacted VAMCs: Houston, Tampa, and San Juan.

The initial study cohort for the Houston VAMC included VA healthcare-users who had accessed the VAMC at least once in the 24 months prior to Hurricane Harvey. Similarly, for the other two VAMCs (Tampa and San Juan), the initial study cohort included VA-users who had accessed their respective VAMC at least once in the 24 months prior to Hurricanes Irma and Maria.

For the Houston and Tampa VAMCs, the total number of daily HBPC services and telephone HBPC visits were examined 14-days before and 14-days after each hurricane. Given that Puerto Rico experienced extensive damages from Irma and Maria, for the San Juan VAMC, the total number of daily HBPC services and telephone HBPC visits were examined 30-days before and 30-days after each hurricane. The percentage of HBPC patients with one, two, three, and four or more HBPC visits were also examined two-weeks before, one-week before, one-week after and two-weeks after each hurricane.

## Results

### Qualitative analysis

Thirty-four respondents, including site program directors and representatives from nursing, social work, occupational therapy, and dietetics, participated in this study. The size of the HBPC programs ranged from a single, small CBOC, with a census of 45 to a large VAMC-centered program, with a census of more than 500, with an overall patient census of 3118 for all nine programs. No program reported a loss of life due to the Hurricanes.

**Fig. 1:**
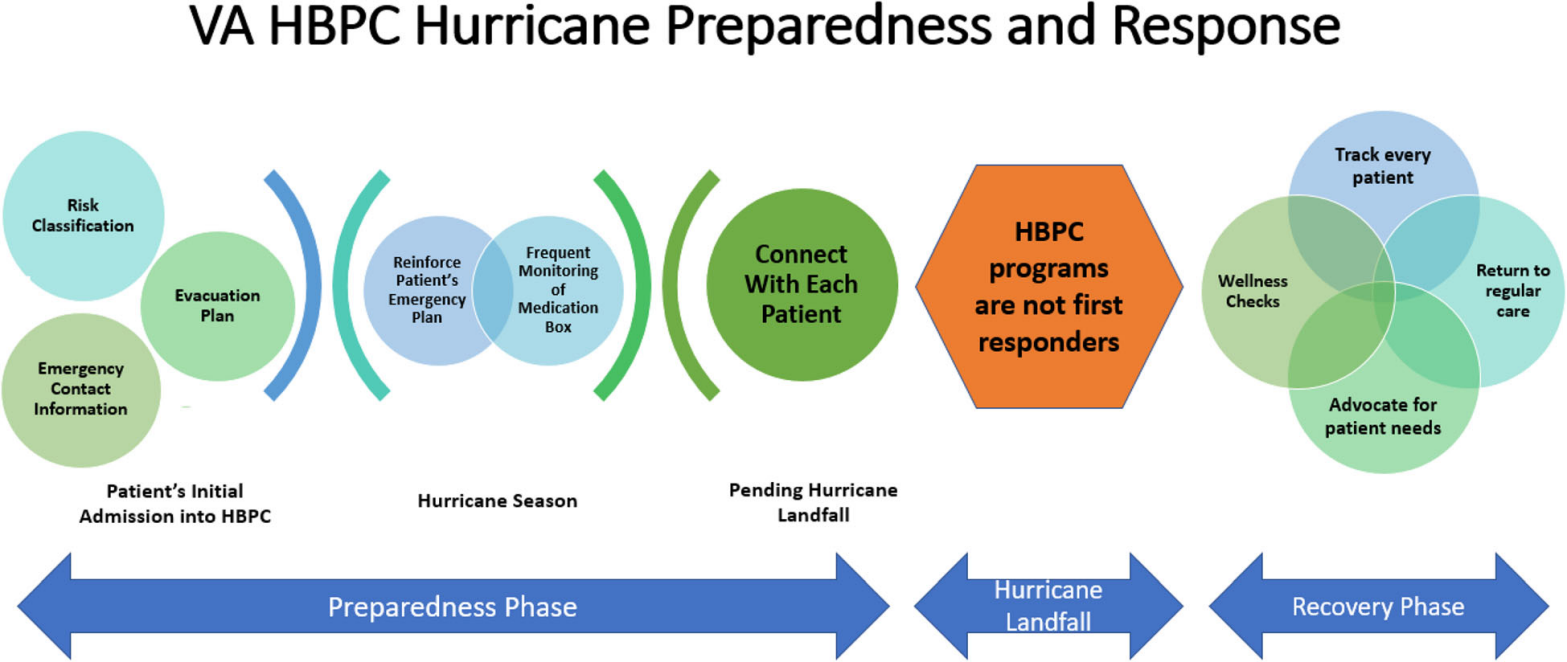
VA HBPC Hurricane Preparedness and Response

The emergency management activities of the HBPC programs emerged as two distinct phases: preparedness, and response and recovery. Both phases, identified in the qualitative data, were supported by the quantitative analysis (see Fig. 1).

#### Preparedness

Preparedness activities in HBPC programs situated in the Hurricane regions start early and have a three-pronged approach. At initial admission, patients are categorized into a risk category group. The risk categorization parameters differ based on the program, but generally include region, device use, and caregiver support. As explained by a Nurse Practitioner at Site 5*, “It’s a risk form that the higher their number the higher the risk, basically. And then we used individual providers [who] know the patients and we can say okay, this persons on oxygen, he lives alone, he’s by the river in a mobile home. That’s high risk*.” Program staff begin by identifying additional caregiver support for the patient and the outlines of an emergency plan. Although this activity is often led by the nursing staff, it can be undertaken by various HBPC team members. At Site 2, social workers oversaw preparedness planning:

Social workers meet with [patients] quarterly. And this is April, by May within the next two months I will have the social workers go through and make sure that our numbers are good. I think it’s one of the biggest things that sometimes the [phone] numbers don’t get updated. I always want to be proactive so that June 1st is the beginning of hurricane season. So by the time June 1st gets here I will be set with what we will be doing and we’ll have an outline plan if something should come up. (Program Director)

Once hurricane season (June 1–November 30) commences, programs enter a heightened level of preparedness. This phase includes constant monitoring of medication boxes, reviewing emergency plans, and supporting patients in signing up for emergency transportation and shelter support. As explained by a Social Worker, *“[We have a] template…And it says what the person’s zone is, what the special needs shelter is, whether we registered them, what their evacuation plan is, and we educate them about what they need to bring if they do evacuate, what to do with their pet”* (Site 4). Finally, program staff underscore with patients that the HBPC program will not act as first responders.

In the event a hurricane landfall is pending, programs move into their final stage of preparedness: calling each patient, checking their status, and verifying their emergency plan. Experienced program directors start this process about 5 days prior to projected landfall, but many sites reported last minute challenges, including needing to encourage patients to evacuate, especially in areas where hurricane landfall projection changed over the course of time. Staff reported that patients who were firmly against evacuation at the beginning of the week became nervous as the hurricane got closer, sometimes waiting until the last day to change their mind and needed assistance to evacuate. Some staff described their role as advocates for community resources to help their clients.

We had some issues with the special needs shelter and the office of emergency planning, so I ended up doing a lot of troubleshooting later in the week regarding issues that that system was having. They tried to pick up one of my gentlemen that’s chair bound with a school bus with no lift. One of my patients that had pre-arrangements for a hospital got turned away at the door…Late Friday [the day before the storm] was getting hairy. Friday I was on the phone with the fire department begging them to extract a 400-pound man that lives in a recreational vehicle who can’t even sit up right that needs to be ambulance transported. The RV is not intended for long term habitation and took a team of people to go get him…So Friday was crazy. It’s not people that could go to a hotel, it’s not people that went to the special needs shelter, it was the highest need people that it wasn’t working properly. (Social Worker, Site 4)

A novice program director explained why waiting until receiving permission from their VAMC to activate their emergency plan was a mistake:

Well, we had contacted the main program down in [location de-identified] because we wanted to start contacting patients as soon as we knew about landfall. So we could reach them. And they said you don’t need to worry about it yet. We usually try to follow directions and so we waited. They decided that we couldn’t start reaching out to patients until about the day before the hurricane. The next time we will not just listen we will go with our gut and do what we need to do because it could’ve been handled much better. But we didn’t start contacting patients until the day before. Maybe a day and a half before. Then we had about a half a day, maybe about six hours trying to get ahold of everybody. Because then we all needed to go home to board up our homes, evacuate if we we’re going to. (Site 1)

#### Response and recovery

Respondents across all sites commented on the speed with which staff initiated these activities once the response began. For sites only marginally impacted by the hurricane, program staff were back in the office the first business day after the hurricane. For programs where the region the CBOC was located was severely impacted but the primary VAMC’s region was not, staff at the VAMC initiated calls to patients immediately after the hurricane.

In more severely impacted regions, staff often needed to go out to patients’ homes to check on them because they were unable to consistently reach patients by phone.

All the staff were asked to visit [the patients]. I'm telling you I had everybody. Social workers, physical therapists, the doctors, every single person we did by visitation. And all they asked us to do is make sure that they were okay. So we didn’t [go] there to do much of intervention. We didn’t have supplies to wrap them either but we went to make sure that they were okay. And if they were not okay to bring him back here to the command center to make sure that the patient is close to the hospital. (Nurse Practitioner, Site 6)

Some staff helped patients who had evacuated understand the status of their home, “*I know for some of the patients who lived in [location de-identified], myself and the doctor would go out and do things for the patients like take pictures of their house so that they would know what the status. One house had a, you know, the roof was partially gone, and the air conditioner was knocked over. So, they could kind of plan and make sure they knew what was going on.”* (RN, Site 1).

The primary goal of the first outreach was a well-visit, as opposed to a regular “clinic” visit. Staff in harder hit areas found this challenging, because they felt helpless to provide support to their patients. This spurred at least one site to work with local relief agencies. As explained by the Program Director, *we worked with some community agency and were able to provide water…so our staff goes to each house with a box of water. Almost 24 bottles of water for them and [the patients] were so happy because they don’t have water.* (Site 6).

### Quantitative analysis

**Fig. 2:**
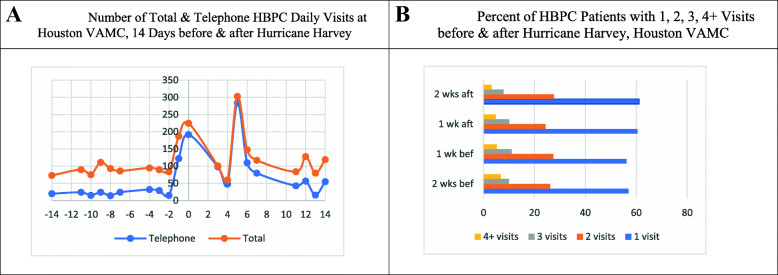
Houston VAMC. **a**. Number of Total & Telephone HBPC Daily Visits at Houston VAMC, 14 Days before & after Hurricane Harvey. **b**. Percent of HBPC Patients with 1, 2, 3, 4+ Visits before & after Hurricane Harvey, Houston VAMC

Fig. 2a displays the total number of HBPC visits, including telephone visits, at the Houston VAMC. One to 2 days before Harvey, the total number of HBPC visits, including telephone visits, increased substantially from an average of 100 daily visits to 225 on the day Harvey made landfall. Four-days post Harvey, the number of HBPC visits decreased to 50 but rapidly increased to over 300 visits the following day when flooding in Houston became a major concern. Figure 2b shows the weekly percent of HBPC patients with one, two, three, and four or more HBPC visits at the Houston VAMC before and after Harvey. Overall, there was a modest change in the percentage of HBPC patients receiving HBPC weekly visits before compared to after Harvey. For example, 56% vs. 60% of HBPC patients had one visit one-week before vs. one-week after Harvey.

**Fig. 3:**
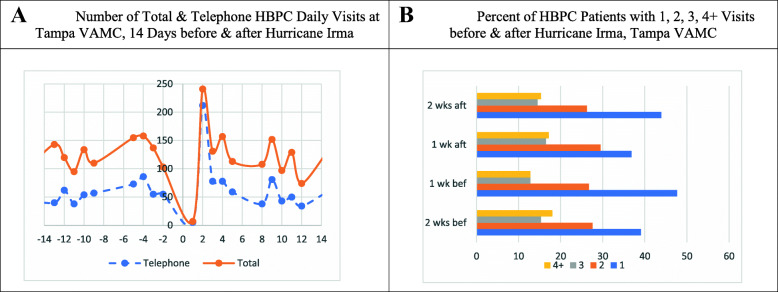
Tampa VAMC. **a**. Number of Total & Telephone HBPC Daily Visits at Tampa VAMC, 14 Days before & after Hurricane Irma. **b**. Percent of HBPC Patients with 1, 2, 3, 4+ Visits before & after Hurricane Irma, Tampa VAMC

Figure 3a displays the total number of HBPC visits and telephone visits at the Tampa VAMC. One week before Irma, the total number of HBPC visits, including telephone visits, increased from an average of 100 daily visits to 150 one-day before Irma made landfall. Two-days post-Irma, the number of HBPC visits increased substantially to 250 but rapidly decreased to pre-Irma levels. Figure 3b shows the weekly percent of HBPC patients with one, two, three, and four or more HBPC visits at the Tampa VAMC before and after Irma. There was a slight change in the percentage of HBPC patients receiving weekly visits before vs. after Irma. For example, 48% vs. 37% of HBPC patients had one visit one-week before vs. one-week after Irma.

**Fig. 4:**
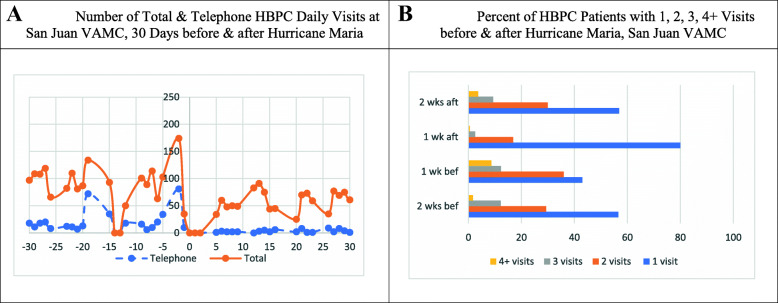
San Juan VAMC. **a**. Number of Total & Telephone HBPC Daily Visits at San Juan VAMC, 30 Days before & after Hurricane Maria. **b**. Percent of HBPC Patients with 1, 2, 3, 4+ Visits before & after Hurricane Maria, San Juan VAMC

Figure 4a displays the total number of HBPC visits and HBPC telephone visits at the San Juan VAMC. Immediately before Irma, the total number of HBPC visits increased from an average of 75 daily visits to 134 on the day Irma made landfall. One-day after Irma, there were no HBPC visits. However, within 3 days after Irma, HBPC visits started to increase, reaching its peak at 174 one-day before Maria made landfall. Immediately after Maria, there were no HBPC visits. Within three-days post Maria, HBPC visits increased, reaching slightly below pre-Irma levels. Figure 4b shows the weekly percent of HBPC patients with HBPC visits at San Juan VAMC before and after Irma. Overall, there was a drastic change in the percentage of HBPC patients receiving HBPC weekly visits before vs. after Irma. For example, 43% vs. 80% of HBPC patients had one visit one-week before vs. one-week after Maria. In contrast, there was a sharp decrease in the percent of patients who received two (36% vs. 17%), three (12% vs. 3%), and four or more (9% vs. 0.5%) HBPC weekly visits one-week before vs. one-week after Maria.

## Discussion

From 2015 to 2030, the percent of individuals age 65 and older worldwide will increase from 9 to 12% [19]. In the United States alone, the number of people 85 years and older is expected to nearly double from 2016 to 2035 [20]. With an increased ability to provide health care to individuals in their home, more older individuals are now choosing to age in their homes. And although there is an increasing evidence base on the value of providing healthcare to patients in their homes [21, 22], there is limited research about how to support these individuals during a disaster. The role of home-based health care programs as critical components in supporting the older old during disasters is becoming clearer [23–25]. However, few studies have examined the activities required by these programs to support a real disaster [26]. Accordingly, we sought to explore the activities conducted in advance of, and in response to, a natural disaster (in this case, hurricanes) and determine the resources required to support such activities. We found that VA HBPC program staff undertook extensive preparedness actions and quickly resumed delivering patient care even in the most impacted regions.

The disaster resilience of place (DROP) model proposes that the total disaster impact on a community is defined through a combination of antecedent conditions [27]. SL Cutter, L Barnes, M Berry, C Burton, E Evans, E Tate and J Webb [26] enumerate several ways in which a community can respond to a disaster event, including predetermined evacuation plans, creation of shelters, information dissemination, and emergency response plans. The overall local impact of a disaster can be moderated by the ability of the community to absorb event impacts using predetermined coping responses [27].

The multiple preparedness phases implemented by the VA’s HBPC programs demonstrate the efforts required to invest in the types of antecedent conditions that enhance a community’s absorptive capacity. Prior research has shown that the older old and their caregivers are often isolated from their communities [28, 29] and unprepared for disasters [8]. By talking with patients about emergency plans, providing information and support for shelter registration, and heightening medication management during the hurricane season, HBPC program staff bridge this essential gap between patients and their communities. This early groundwork allows the HBPC programs to call each patient and support execution of the pre-established emergency plan. Without the initial groundwork, the management of an entire census of patients would be much more challenging.

Moreover, these three phases of preparedness set up the possibility of a rapid response if required. As shown by SA Bell, J Horowitz and T Iwashyna [24], the response period was quite challenging for most HHAs after Hurricane Harvey. In contrast, we found minimal delays in following up with patients after the hurricane events. In cases where patients had evacuated, staff reported on the status of their patients’ homes, and even in Puerto Rico, the hardest hit region, our data show program staff beginning to conduct well-visits within 3 days of landfall. These findings exemplify the vital role of the HBPC program in bolstering community resilience.

Past disasters have demonstrated that the majority of medical surge arises from individuals with one or more chronic conditions or dependency on electric-dependent equipment [7, 9, 30–32]. Understanding ways to reduce this medical surge in the days after a disaster is an essential piece to bolstering a region’s disaster resilience [33]. A well-visit conducted by skilled clinicians who are familiar with their patients’ care, can provide essential support to medically vulnerable individuals and potentially mitigate some of this surge. Indeed, our study showed that those patients who prior to the hurricane were only being seen once a week, did not require additional visits. In addition, both the qualitative and quantitative data demonstrate that the temporary reduction in care for higher frequency patients did not result in any critical disturbance to patients’ medical status in the immediate post-hurricane period. These findings highlight the significance of intense preparedness and a structured response.

As our cities grow, and the share of residents aged 60 and older steadily increases, more resources need to be dedicated to understanding how to support active aging and aging in place. Specifically, communities need to expand their conversation about how to better support active aging by optimizing opportunities for health and security [34, 35]. One way in which disasters exacerbate existing deficiencies is by stressing existing infrastructure. The vulnerability of older adults after disasters is in part related to a lack of existing social support before a disaster event. As has been noted in our results, home-based care programs can bolster socially isolated, homebound individuals support structures, yet they cannot do it alone. As has been noted in the literature, although the larger field of HHAs have an important role to play in community disaster response [23], in practice, few agencies are part of local healthcare coalitions, a key center of local disaster response [24]. Understanding HHA preparedness and response activities within the context of an actual disaster can help healthcare coalitions understand the need to and potential benefits of incorporating HHAs into such coalitions and their response strategies, and by extension provide increased attention and support to our growing population of older adults.

### Limitations

This study has limitations. First, the data collection and design of the qualitative and quantitative components were conducted independently. Future studies should incorporate a more integrated mixed methods approach where one method (qualitative or quantitative) guides the other. Second, the findings from the interviews cannot be generalized to all VA HBPC programs. Third, quantitative data were reported by staff via electronic health records. Future studies should incorporate data from disaster-impacted patients. Finally, the VA HBPC program is a singular example of home-based care programs and has the support of the larger VA healthcare system during emergencies. Nevertheless, VA HBPC programs’ activities are examples of best practices. Future studies should examine whether (a) programs located in disaster-prone regions are more prepared than others, (b) how to ensure adequate preparedness in regions that are less frequently impacted by disasters, and (c) how non-VA home-based care programs support their patients and staff during emergencies.

## Conclusions

Historically, caring for the older old has been a challenge in the aftermath of disasters due to the limited presence of pre-established coping mechanisms. And as our population ages, and aging in place becomes more common, more attention needs to be placed on how to support our older old, particularly those with medical vulnerabilities, around disasters. Home based care programs such as HHAs and the VA’s HBPC program, which already have strong relationships with their patients and help connect homebound, medically complex, older old to the greater healthcare community, can play an essential role in establishing and fulfilling these coping mechanisms. Engaging with these programs both pre- and post-disasters is central to bolstering community resilience for these at-risk populations.

## Supplementary Information

**Additional file 1: Supplementary file 1.** Fall 2017 Hurricanes Interview Guide. Semi-structured interview guide to guide data collection.

## Data Availability

The datasets used and/or analyzed during the current study are available from the corresponding author on reasonable request.

## Abbreviations

VA: US Department of Veterans Affairs
HBPC: Home Based Primary Care
CDW: Corporate Data Warehouse
HHA: Home health agencies
CMS: Centers for Medicare and Medicaid Services
IAH: Independence at Home
CBOC: Community Based Outpatient Clinic
VAMC: VA Medical Center
RV: Recreational vehicle
DROP: Disaster resilience of place
