# Supplementary material for: Preparedness and response activities of the US Department of Veterans Affairs (VA) home-based primary care program around the fall 2017 hurricane season
Source: BMC Public Health. 2020 Nov 26;20:1796. doi: 10.1186/s12889-020-09888-8 (PMC7690102; doi:10.1186/s12889-020-09888-8)
Supplement: Supplementary file 1 — Additional file 1: Supplementary file 1. Fall 2017 Hurricanes Interview Guide. Semi-structured interview guide to guide data collection. [file 12889_2020_9888_MOESM1_ESM.docx]

**Introduction**

This study is being conducted by researchers from the VA Greater Los Angeles Healthcare System and has been approved by its IRB. Our goal is to gain a better understanding of the disaster response for veterans in the VA’s Home Based Primary Care program following Hurricanes Harvey, Irma, and Maria. All activities are being undertaken for research purposes only. All information that we collect will remain confidential to the fullest extent allowed by law. We will not disclose identifying information concerning you, other staff, or patients. Participation in this project is entirely voluntary and will not affect your employment. You may refuse to answer any question or stop participating at any point in the research. The entire interview should take about an hour. There is no right or wrong answer to these questions. We are interested in your experiences and opinions. Do you have any questions thus far?

**Verbal Consent:** “Thank you for agreeing to participate in this interview. Before we begin, it is necessary that I get your verbal consent to audiotape this focus group. This recording will only be used for this study. Do I have your consent to audiotape you during this interview? Please say yes to indicated that you are giving your permission for this audio recording”.

**The first few questions will ask you a bit about your position with HBPC.**

1. What is your position with the HBPC program?
   1. Did you have a different position during the time of [the relevant disaster activity period]?
   2. If yes – What was your former position?
2. Is your role clinical, administrative, or both?
   1. What are the main types of activities that you are involved in with the HBPC program?
   2. Is your role different now than before [the relevant disaster activity period]?
3. How many years have you been with the HBPC program?
4. Have you ever weathered an emergency like this before as a part of an HBPC program? Had your site ever participated in a full scale public health emergency?
5. What disaster preparedness training have you received as an HBPC practitioner?

**BACKGROUND QUESTIONS FOR PROGRAM DIRECTORS ONLY**

1. Approximately how many patients did you have on your census in a typical month before [the relevant disaster activity period]?
2. How many patients did you have on your census now?

**CONTINUE INTERVIEW FOR ALL RESPONDENTS**

**For the next section of questions, we’d like you to specifically think about disaster assessment protocols and procedures undertaken in advance of Hurricane Harvey/Irma/Maria.**

1. When were you advised of the coming Hurricane, and by whom?
2. Who was responsible for coordinating the pre-Hurricane preparedness steps?
3. Just prior to [the relevant disaster activity period], did you receive any targeted disaster preparedness training?
4. What steps were taken by your program in advance of the coming [relevant disaster activity period]? Including, but not limited to:

a. Contacting staff

b. Contacting patients

- 1. Was there a pre-assessed level of preparedness for their patient population (i.e. risk categorization)?
  2. Was any specific screening tool used (e.g., HBPC Toolkit, acuity scale, GIS mapping, patient disaster assessment tool) to identify those at highest need for assistance because of [the relevant disaster activity period]?

c. Helping patients decide if they should shelter-in-place or evacuate

- Securing information on shelter options for patients and staff
- Did your parent VA support you in providing these resources?
- What factors were involved in this decision-making process?
- Caregiver availability/willingness to stay

1. For those patients for whom you recommended evacuation, did you get any resistance from them or their family members? If so, how did you deal with this?

e. Were there any last-minute medication management issues?

**Next, we’d like to ask you about procedures undertaken during the immediate period of [the relevant disaster activity period] as well as the short-term aftermath.**

1. How did you deal with staffing over the course of [the relevant disaster activity period]?

a. How often were you in contact with your staff during the storm and subsequent rain fall?

2. Did any of your staff shelter in the hospital?

3. Was sheltering at the hospital an option available for patients?

If yes: What was the process for accepting and hosting patients?

How many patients sheltered in the hospital? For how long?

Did you bring patient’s families/caregivers as well?

4. What were some of the situations patients found themselves in such as evacuating or sheltering in place?

5. How were you keeping track of your patients?

6. What aspects of [the relevant disaster activity period] were complicated by the patient conditions such as physical, cognitive or behavioral problems?

7. What provisions were made to ensure continuity of care surrounding the [the relevant disaster activity period] and the subsequent two weeks?

**Let’s turn to procedures undertaken during the period after [the relevant disaster activity period].**

1. Aside from the services you regularly provide, what additional assistance are you providing to your patients?
   1. E.g, Are you assisting residents with permanent placement/relocation?

**We are almost to our last section of questions. This section will focus on actions taken by the HBPC program(s) as part of “after action reports” and evaluation based on lessons learned.**

1. How have written policies concerning how your program should manage its patients during disasters changed since [the relevant disaster activity period]?

*Probes:*

1. Did someone tell you to change or did your facility change?
2. Are there are any planned changes in the works? Who planned these changes and why?
3. Do you anticipate that unplanned changes might occur? Why?
4. How quickly do you think policy will change? Over what time period? Why?

2. How have types of in-services and other training experiences about public health emergencies (evacuation-specific or otherwise) available to your staff changed since [the relevant disaster activity period]?

*Probes:*

- What is available?
- Who attends?
- How often does staff attend?
- What type of staff training do you think is needed and for whom?
- Are the subjects covered different?

**Lastly, we want to know about any other lessons learned that we haven’t already discussed.**

1. Have you communicated with other HBPC programs or home health agencies since [the relevant disaster activity period]? If so, what did you learn?

4. What could your VA medical center/VACO change to improve disaster preparedness for HBPC patients?

4. How could we improve the care for patients during these types of disasters?

5. What about [the relevant disaster activity period] surprised you (e.g., either how well it went or unexpected difficulties that arose)?

6. What else would you like to tell us about dealing with disaster preparedness and veterans surrounding [the relevant disaster activity period]?

**Before we conclude the interview, do you have any suggestions for others we might be able to contact?**

Who else should we be sure to talk to who knows a lot about the experience of HBPC programs due to [the relevant disaster activity period]? How shall we contact him/her?
